# The effects of Tissue-type Plasminogen Activator on PSC Activation

**DOI:** 10.2174/0118715303394327251127145651

**Published:** 2026-04-24

**Authors:** Min Zha, Yi Wei, Shu Zhang

**Affiliations:** 1 Department of Endocrinology, Jiangsu Province Hospital of Chinese medicine, Affiliated Hospital of Nanjing University of Chinese Medicine, Hanzhong Road, Nanjing, China

**Keywords:** Pancreatic stellate cell, tissue-type plasminogen activator, activation, type 2 diabetes mellitus, clinical research, extracellular matrix

## Abstract

**Introduction:**

Our previous studies have demonstrated that the activated pancreatic stellate cell (PSC) could induce islet damage in type 2 diabetes mellitus (T2DM). While tissue-type plasminogen activator (tPA) is significantly reduced in T2DM, its subsequent effects are unclear. The purpose of this experiment was to observe the impact of tPA on PSC activation, with the aim to better understand the potential role of tPA in T2DM.

**Methods:**

50 type 2 diabetic patients and 50 healthy persons were included in the diabetic group and the control group, respectively. Fasting blood was collected separately, and the tPA-level was detected by ELISA. Rat PSCs were isolated from pancreatic tissue using standard explant techniques. The PSCs were then characterized by staining them with Oil Red O to visualize lipid droplets and using immunofluorescent markers (α-smooth muscle actin (α-SMA), vimentin, and glial fibrillary acidic protein (GFAP)). After characterization, the PSCs were treated with tPA, then the proliferation of PSCs was measured using the cell counting kit-8 (CCK-8), the apoptosis was observed by the caspase-3 fluorometric assay kit, and the migration ability was assessed using the wound-healing assay and the transwell migration assay. Finally, a Western blot was used to identify the extracellular matrix (ECM) component synthesized by PSCs.

**Results:**

The diabetic patients had significantly lower levels of tPA compared to the controls. Rat PSCs treated with tPA exhibited more lipid droplet accumulation, and their ability of proliferation, migration, and ECM synthesis were significantly inhibited.

**Discussion:**

These findings suggest that tPA may serve as an endogenous suppressor of PSC activation, and its deficiency in T2DM likely contributes to islet fibrosis and progressive β-cell dysfunction.

**Conclusion:**

This study demonstrated that tPA can play a crucial role in significantly inhibiting the activation, proliferation, migration, and ECM synthesis of PSC. Therefore, we speculate that the significant reduction of tPA in T2DM may exacerbate the detrimental effect of PSC on *β*-cell function.

## 1 INTRODUCTION

The central role of activated pancreatic stellate cells (PSC) in promoting the development of fibrosis in pancreatic diseases such as chronic pancreatitis, pancreatic cancer has been confirmed. And our previous studies have demonstrated that PSC activation induces islet damage such as beta-cell failure and islet fibrosis, which plays a crucial role in the progressive deterioration of *β*-cell function associated with type 2 diabetes mellitus (T2DM) development [1-4]. Although the specific mechanism of PSC activation is not yet fully understood, identifying its activation factors and mastering its activation mechanism will be crucial in developing new therapeutic targets to combat pancreatic diseases.

We have also observed that high glucose aggravates the detrimental effects of PSC on beta-cell function [4]. Other pathologic stimuli associated with T2DM can also lead to PSC activation. It was reported that plasminogen activator inhibitor-1 (PAI-1) deficiency reduced hepatic fibrosis after bile duct obstruction mainly through the activation of tPA and hepatocyte growth factor (HGF) [5]. This suggests that tPA can reduce liver fibrosis. However, it is still unknown whether tissue-type plasminogen activator (tPA) has a similar effect on pancreatic fibrosis or even islet fibrosis in T2DM. Previous research showed that tPA is significantly reduced in T2DM. If tPA does have such an effect on pancreatic fibrosis or even islet fibrosis in T2DM, it may impact the activation of PSC. Therefore, the purpose of this experiment was to investigate the potential relationship between tPA and PSC activation in order to better understand the role of tPA in T2DM.

## 2 MATERIALS AND METHODS

### 2.1 The Diabetic Patients and the Experimental Animals

According to the diagnostic criteria of T2DM formulated by the World Health Organization in 1999, 50 patients with T2DM admitted to our hospital between January 2020 and April 2023 were recruited in the diabetic group. The age of the patients was (34-76) years old, with an average age of 56.88 years old, excluding diseases of the liver, kidney, tumor and blood system; The control group consisted of 50 healthy physical subjects, aged 35-76 years old, average 55.96 years old (no significant difference in age and sex compared with the diabetic group). The subjects had not taken any medication or diet that had effects on the coagulation system during the two weeks prior to the blood draw. 2ml of fasting venous blood was taken from the subjects in the morning and placed in an anticoagulant tube containing sodium citrate, mixed and centrifugated (3000 r/min, 5min), the plasma was separated in time. ELISA was used to identify tPA, and the kit was purchased from GILED biotechnology.

Wistar rats were purchased from the Shanghai Slaccas Laboratory Animal Center, with body weights ranging from 270 to 310 grams. The rats were housed four per cage. Following a one-week acclimation period, these 8-week-old rats were utilized for experiments.

This study was approved by the ethical review committee of Jiangsu Province Hospital of Chinese Medicine (2018NL-108-02 for clinical research and QK-20200408-001 for animal research) and conducted in accordance with the Declaration of Helsinki. Each participant provided written informed consent.

### 2.2 Isolation, Culture, and Characterization of PSC

The methods were introduced in the previous study [6, 7].

### 2.3 Oil Red O Staining

PSCs were fixed at room temperature for 25 minutes using PFA. To visualize lipid accumulation, Oil Red O (Sigma) staining was performed by incubating the PFA-fixed samples in a saturated solution of Oil Red O dissolved in isopropanol for 10 minutes. After staining, the samples were thoroughly washed with distilled water to remove excess dye. Images were captured using phase-contrast microscopy (Zeiss), ensuring clear visualization of lipid droplets.

### 2.4 Immunostaining

PSCs were fixed and subsequently subjected to immunofluorescent staining for α-SMA, vimentin, GFAP, collagen I (Col-I), collagen III (Col-III), and fibronectin (FN). Cells were incubated overnight at 4°C with primary antibodies (Sigma) at dilutions of 1:1000 for α-SMA, vimentin, and GFAP, and 1:500 for Col-I, Col-III, and FN. The following day, cells were treated with a secondary antibody (Jackson Immunoresearch Laboratories) at a 1:150 dilution for 1 hour. Morphometric analyses were performed using ImageJ software to assess fluorescence intensity and cellular structure.

### 2.5 CCK-8 Assay

Cell proliferation was determined with the CCK-8 kit (Keygen Biotech). The PSCs were suspended at a final concentration of 2 ×10^3^ cells/well. The cells of the control group were incubated in DMEM/F12 with 10% FBS, and the tPA group was supplemented with an additional 10nM tPA. After 12, 36, and 72 hours of incubation, 10 μl of the CCK-8 reagent was added to each well, followed by an additional 1-hour incubation at 37°C. Cell viability was assessed using an automatic microplate reader (Wellscan) by measuring absorbance at 450 nm (A450 nm), where the A450nm value was directly proportional to the rate of cell proliferation. All experiments were conducted in quintuplicate and repeated three times independently (n = 15).

### 2.6 Caspase-3 Assay

A Caspase-3 cell apoptosis experiment was conducted using P5-generation PSCs. The control group was cultured in serum-free medium, while the experimental group was supplemented with an additional 10 nM tPA. After 24 h, caspase-3 apoptosis reagent (Abcam) was added, and the fluorescence intensity was measured (excitation wavelength 380nm, emitted light wavelength 430-460 nm). Each group contained 8 wells, and the experiment was repeated independently three times.

### 2.7 Cell Migration Assays

The transwell migration assay was conducted in 24-well transwell plates using inserts with an 8 μm pore size (Corning). A total of 5×10^3^ pancreatic stellate cells (PSCs) were serum-starved for 24 hours before being seeded into the inner compartment of the transwell insert in serum-free medium. In the tPA group, an additional 10 nM tPA was supplemented. The lower wells of the plate contained DMEM/F12 medium supplemented with 10% FBS to serve as a chemoattractant. After 24 hours of incubation at 37°C, cells were fixed with 2% glutaraldehyde and stained with 1% crystal violet for 10 minutes. Non-migrated cells on the upper membrane surface were removed, while cells that had migrated to the lower side of the membrane were visualized under a light microscope and counted manually (n=5).

For the wound-healing assay, 5×10^5^ PSCs were seeded into 6-well culture plates and grown to confluence. After serum starvation for 24 hours, a wound was created by scraping a strip of cells using a 10 μL pipette tip. The control group was incubated in DMEM/F12 medium with 10% FBS, while the tPA group received an additional 10 nM tPA. After 24 hours, the number of cells that had migrated into the wound area was manually counted under a phase-contrast microscope (n=5).

### 2.8 Western Blot Analysis

The cells of the control group were incubated in DMEM/F12 with 10% FBS for 24 h, while the tPA group was supplemented with an additional 10nM tPA. The total protein of PSCs was extracted using RIPA buffer containing protease inhibitors. The whole-cell lysates protein concentration was quantified, 50 μg was subjected to SDS–PAGE then blotted onto a nitrocellulose membrane. The membranes were blocked in a buffer containing 5% non-fat dry milk dissolved in Tris-buffered saline with Tween 20 (TBST), then incubated overnight with primary antibodies against Col-I/III and FN (Abcam, 2µg/ml) and GAPDH (Abcam) as an internal control, dilution 1:1000. After that, the membranes were washed 5 times prior to incubation with HRP-conjugated secondary antibody for 1 h. The blot was developed using a super-sensitive ECL Chemiluminescence Kit.

### 2.9 Statistical Analysis

Data were presented as the mean ± SE. Statistical significance was determined by t-test, as appropriate, and differences between groups were considered to be statistically significant when *P* < 0.05. All of the statistical analyses were performed using the Statistical Product and Services Solutions (SPSS) package (Version 11.5, SPSS Science).

## 3 RESULTS

### 3.1 The tPA Levels were Significantly Lower in the T2DM Patients

There were no significant differences in age or sex between the diabetics and the controls, but tPA level was significantly lower in the diabetic group compared to the control group (Table **1**), suggesting a significant reduction in tPA in T2DM.

### 3.2 Isolation, Culture, and Characterization of PSC

PSCs were isolated by the pancreatic tissue block culture method. After inoculating the pancreatic tissue blocks in Corning's conventional cell culture dish and placing them in the incubator, PSCs could be seen growing externally from the surrounding adherent pancreatic tissue blocks under the microscope after 48-72 hours, demonstrating a static polygonal state with cytoplasm containing crystal-clear lipid droplets (Fig. **1a**). Following a week of pancreatic tissue culture, PSCs gradually increased and formed a radiating pattern around the pancreatic tissue. The primary cultured PSCs were still in a quiescent state, and their typical feature was the presence of lipid droplets rich in vitamin A in the cytoplasm, which was confirmed by oil red O staining (Figs. **1b** and **c**). Classical identification markers, such as α-SMA, GFAP, and vimentin, were used to purify PSC by pancreatic enzyme digestion and passage. Immunofluorescence staining was performed on the 3rd generation of PSC that were progressively activated by passage. The expressions of α-SMA (Fig. **1d**), vimentin (Fig. **1e**), and GFAP (Fig. **1f**) were observed in the PSCs.

### 3.3 Effect of tPA on Plasma Lipid Droplet Content in PSC

After 7 days of pancreatic tissue culture, a significant amount of PSC was found around the pancreatic tissue, with lipid droplets rich in the PSC cytoplasm colored in crystal orange-red by oil red O staining (Fig. **1b** and **c**). The addition of tPA into the tissue block culture medium significantly increased the content of lipid droplets in PSC (Figs. **1g** and **h**).

### 3.4 Effect of tPA on PSC Cell Proliferation

In order to observe the effect of tPA on PSC proliferation, the absorbance values at 450 nm, which are proportional to the number of cells, were detected by the CCK-8 kit for both the control and the tPA intervention group. As shown in Fig. **2**, the PSCs were implanted into a 96-well plate after digestion by pancreatic enzymes. After incubation overnight for 12 h, the PSCs were attached to the wall. At this point, there was no statistically significant difference in absorbance values between the control and the tPA intervention group, indicating that there was no statistically significant difference in the number of cells between the two groups prior to the intervention. After the intervention of tPA, the absorbance value of the tPA intervention group decreased significantly after 48 h compared with the control group, with statistical difference (*P*<0.01), and the value of the tPA intervention group further decreased significantly after 72 h (*P* <0.01), indicating that the use of tPA led to a significant decrease in the number of PSC when used *in vitro* experiments.

### 3.5 Effect of tPA on PSC Apoptosis

After being treated with a 48-hour exposure to tPA, the apoptosis level of PSCs was detected using a fluorescence Caspase-3 activity detection kit. The result showed that the fluorescence value of the tPA group was significantly higher than that of the control group (*P* <0.05, Fig. **3**), indicating that tPA could induce apoptosis of PSCs during *in vitro* culture. ​The findings of this study support the notion that tPA has a detrimental effect on PSCs *in vitro*. One possible explanation for this effect is that tPA may interfere with the apoptotic pathway. However, further studies are needed to confirm this hypothesis.

### 3.6 Effect of tPA on PSC Migration

The migration ability of PSCs under the treatment with tPA was examined in a wound-healing assay and transwell migration assay, both of which are commonly used methods for studying cell migration.

In the wound-healing assay, cell-free areas were formed by scratching the culture plate surface with a pipette tip (Fig. **4a**-**b**), and the number of PSCs migrating into the scratch area after 24 hours was photographed and counted. As shown in Fig. (**4**), PSCs in the control group achieved almost 50% confluence (Fig. **4c**) after 24 hours. However, the migration of PSCs into the cell-free region was significantly decreased in the tPA group (Fig. **4d**) (*P* <0.01).

In addition, the transwell migration assay was also conducted to observe the migration ability of the two groups. In the transwell system, as shown in Fig. (**4e** and **f**), the stained cells appeared purple, while the small black dots represented the pores in the transwell membrane. The PSCs migrated from the upper chamber to the lower chamber through the pores and then attached to the underside of the membrane. The number of PSCs passing through the membrane in the tPA group (Fig. **4f**) was significantly lower than that in the control group (Fig. **4e**) (*P* <0.01).

### 3.7 Effect of tPA on ECM Synthesis of PSC

In this study, we investigated the effect of tPA on the synthesis of ECM components, such as Col-I, Col-III, and FN of PSCs. After the activation of PSCs, large amounts of ECM components can be synthesized and secreted, which contribute to the development of pancreatic fibrosis and subsequent pancreatic diseases. Therefore, we evaluated the expression of the ECM components in PSCs after tPA intervention by Western blot.

The results of this study showed that the expression of Col-I, Col-III, and FN was significantly down-regulated after tPA intervention compared with the control group (*p*<0.01, *p*<0.05, and *p*<0.05, respectively, Fig. **5**). These findings suggest that tPA could inhibit the synthesis and secretion of ECM components in PSCs.

## 4 DISCUSSION

T2DM is a clinical syndrome characterized by elevated blood glucose caused by a combination of insulin resistance and progressive failure of insulin secretion by the *β*-cells in pancreatic islets of Langerhans. However, the cellular mechanisms underlying *β*-cell failure in T2DM have not been well understood. Our previous studies have demonstrated that activation of PSCs could induce islet damage. In addition, high glucose levels could further exacerbate the detrimental effects of PSCs on *β*-cell function, ultimately contributing to the progressive deterioration of *β*-cell function associated with the development of T2DM [4]. Therefore, inhibiting PSCs activation could be a promising approach to treat type 2 diabetes.

In this study, the effect of tPA on the activation of primary PSCs was evaluated through cell experiments. Firstly, primary PSCs were isolated, cultured, and identified *in vitro*. Since specific indicators for PSC expression have not been found yet, PSC was typically identified by the presence of lipid droplets, α-SMA, vimentin, and GFAP. During the *in vitro* isolation and culture process, P0 PSCs generally remained in a quiescent state, characterized by orange-stained lipid droplets using oil red O staining. After passage, PSCs were activated due to trypsin action, mechanical stimulation, and changes in the *in vitro* environment, leading to the disappearance of lipid droplets. The expression of α-SMA, vimentin, and GFAP indicated this transition from a quiescent to an activated state. In this study, tPA was found to reduce the disappearance of lipid droplets in the cytoplasm of PSCs, suggesting that tPA could slow down the process of PSCs activation. Quiescent PSCs can maintain the balance between synthesis and degradation of ECM. Once activated, PSCs synthesize and secrete a large amount of ECM, resulting in excessive ECM deposition and fibrosis. In this study, tPA was found to inhibit the activation, proliferation, and migration of PSC, promote apoptosis, and inhibit the synthesis and secretion of ECM, indicating its potential involvement in the regulation of PSC-induced *β*-cell failure in T2DM.

The plasminogen activators (PAs) are multifunctional serine proteases involved in fibrinolysis, cellular migration, growth factor activation, and tissue repair [8]. Recent studies have shown that tPA is a pro-fibrotic regulator of the MFB phenotype in the kidney [9, 10]. Moreover, the liver also contains HSCs, which are similar to pancreatic stellate cells. HSCs are crucial for liver function as they produce extracellular matrix and regulate tissue repair [11, 12]. Additionally, HSCs are involved in the development of liver fibrosis, a condition characterized by excessive accumulation of fibrous tissue in the liver. The activation and trans-differentiation of HSCs into myofibroblast (MFB)-like cells play a key role in liver fibrosis after injury and represent a potential therapeutic target [9]. In contrast to kidney MFBs, exogenous tPA suppressed activation markers in HSCs instead of inducing them, which is consistent with our findings.

Previous studies have shown that low-density lipoprotein receptor-related protein 1 (LRP1) signaling plays a role in the effect of tPA on HSC-induced fibrosis [13]. Kang's research team experimentally identified t-PA as the signaling ligand for HSC LRP1 and characterized the t-PA/LRP1 ligand-receptor pair on activated hematopoietic stem cells as a novel therapeutic target for anti-liver fibrosis treatment. Furthermore, the tPA-LRP1 pathway was found to play a pivotal role in multiple tumorigenic processes. The Salama team identified the tPA-LRP1 pathway as a key switch driving melanoma progression, partly by regulating the cellular and proteolytic composition of the tumor microenvironment [14]. Qiu *et al.* revealed that the tPA/LRP1-NFκB signaling cascade plays a vital role in driving luminal breast cancer cells [15]. However, it remains unclear whether LRP1 signaling is relevant to tPA-inhibited PSC activation. Therefore, further research is needed.

This study builds upon previous research. In earlier work, the authors described methods for isolating PSC cells without delving into the mechanisms of PSC cell activation [6, 7]. In this study, the authors conducted a clinical observational study revealing elevated tPA expression levels in T2DM patients and further investigated the effects of tPA on the activation of primary PSCs. However, this study has several limitations. We did not employ agonists or antagonists to further validate the implicated signaling pathways, which diminishes the persuasiveness of our findings.

## CONCLUSION

In conclusion, tPA exerts diverse effects on PSCs activations, with potential implications in the management of *β*-cell failure in T2DM. However, further research is needed to elucidate the underlying mechanisms and explore the therapeutic potential of tPA-based interventions. Future studies should also investigate the possible adverse effects of tPA treatment and optimize its dosage and administration in order to maximize its clinical benefits.

## Figures and Tables

**Fig. (1):**
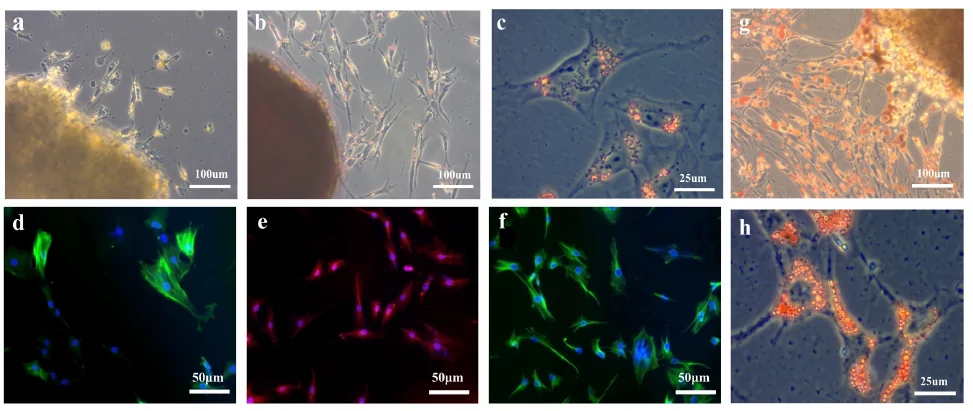
Isolation, culture and characterization of PSCs. (**a**) PSCs grew externally from the surrounding adherent pancreatic tissue blocks after 48-72 hours. (**b-c**) The primary cultured PSCs contain lipid droplets rich in vitamin A in the cytoplasm, which was confirmed by oil red O staining. (**d-f**) The immunofluorescent staining for α-SMA, vimentin, GFAP respectively. (**g-h**) The addition of tPA significantly increases the lipid droplet content in PSCs.

**Fig. (2):**
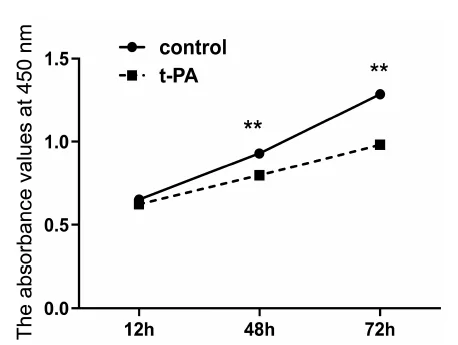
Effect of tPA on PSC proliferation. After the intervention of tPA, the absorbance value of the tPA intervention group decreased significantly after 48 h and 72 h (both **p<0.05).

**Fig. (3):**
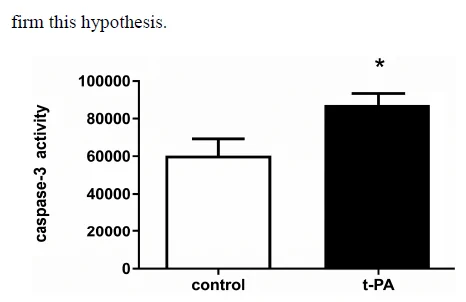
Effect of tPA on PSC apoptosis. After being treated with a 48-hour exposure of tPA, the fluorescence value of tPA group was significantly higher than that of control group (*p<0.05).

**Fig. (4):**
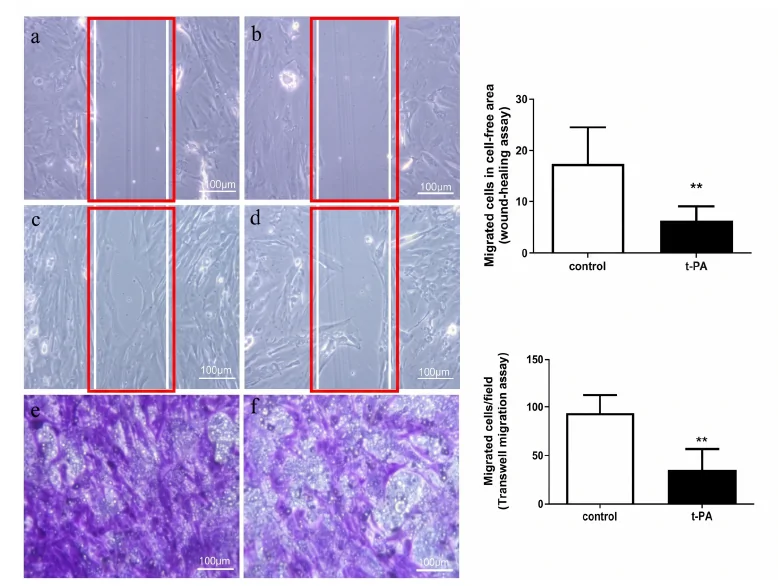
Effect of tPA on PSC migration. The red box indicates the area of the cell scratch. In the wound-healing assay (**a-d**), cell-free areas were formed by scratching the culture plate surface with a pipette tip (**a**) the control group, (**b**) the tPA group), However, compared to the control group (**c**), after 24 hours the migration of cells into the cell-free region was significantly decreased in the tPA group (**d**) (**p<0.01). In the transwell migration assay (**e-f**), The number of PSC passing through the membrane in the tPA group (f) was significantly lower than that in the control group (**e**) (**p<0.01).

**Fig. (5):**
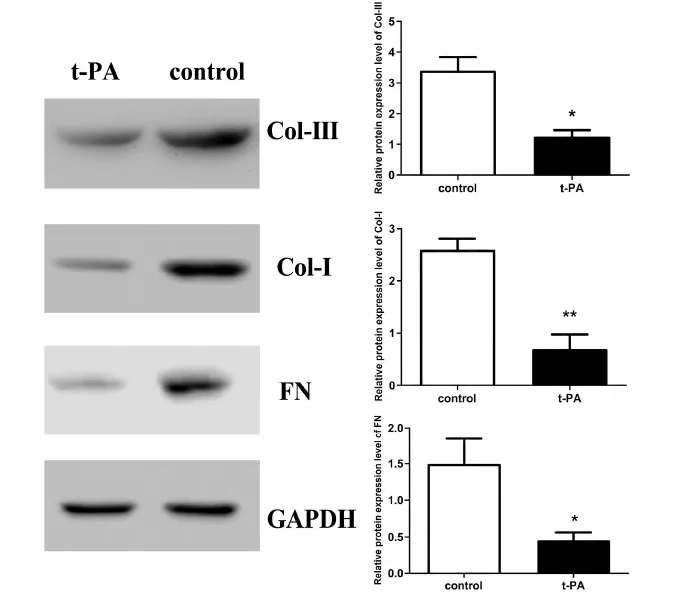
Effect of tPA on ECM Synthesis of PSC. The expression of Col-I, Col-III, and FN was significantly down-regulated after tPA intervention compared with the control group (**p<0.01, *p<0.05 and *p<0.05, respectively).

**Table 1:** tPA level was significantly lower in the diabetic group compared to the control group (*P*<0.01).

-	Age (Year)	Gender (Female/Male)	tPA (ug/mL)
Control	56.88±12.39	25/25	12.26±2.36
DM	55.96±10.45	25/25	9.82±3.33
*P* value	*P*>0.05	*P*>0.05	*P*<0.01

## Data Availability

The data and supportive information are available within the article.
